# Transcranial optical monitoring for detecting intracranial pressure alterations in children with benign external hydrocephalus: a proof-of-concept study

**DOI:** 10.1117/1.NPh.9.4.045005

**Published:** 2022-11-17

**Authors:** Federica Maruccia, Susanna Tagliabue, Jonas B. Fischer, Michał Kacprzak, Santi Pérez-Hoyos, Katiuska Rosas, Ignacio Delgado Álvarez, Juan Sahuquillo, Turgut Durduran, Maria A. Poca

**Affiliations:** a Vall d’Hebron Barcelona Hospital Campus, Vall d’Hebron Research Institute, Neurotraumatology and Neurosurgery Research Unit, Barcelona, Spain; b ICFO-Insitut de Ciències Fotòniques, The Barcelona Institute of Science and Technology, Barcelona, Spain; c HemoPhotonics S.L., Barcelona, Spain; d Nalecz Institute of Biocybernetics and Biomedical Engineering, Warsaw, Poland; e Vall d’Hebron Research Institute, Statistics and Bioinformatics Unit, Barcelona, Spain; f Vall d’Hebron Hospital Universitari, Vall d’Hebron Barcelona Hospital Campus, Department of Neurosurgery and Pediatric Neurosurgery Unit, Barcelona, Spain; g Vall d’Hebron Hospital Universitari, Vall d’Hebron Barcelona Hospital Campus, Department of Pediatric Neuroradiology, Barcelona, Spain; h Universitat Autònoma de Barcelona, Barcelona, Spain; i Institució Catalana de Recerca i Estudis Avançats, Barcelona, Spain

**Keywords:** benign enlargement of subarachnoid spaces, hydrocephalus, intracranial pressure monitoring, optical techniques, pathophysiology

## Abstract

**Significance:**

Benign external hydrocephalus (BEH) is considered a self-limiting pathology with a good prognosis. However, some children present a pathological intracranial pressure (ICP) characterized by quantitative and qualitative alterations (the so-called B-waves) that can lead to neurological sequelae.

**Aim:**

Our purpose was to evaluate whether there were cerebral hemodynamic changes associated with ICP B-waves that could be evaluated with noninvasive neuromonitoring.

**Approach:**

We recruited eleven patients (median age 16 months, range 7 to 55 months) with BEH and an unfavorable evolution requiring ICP monitoring. Bedside, nocturnal monitoring using near-infrared time-resolved and diffuse correlation spectroscopies synchronized to the clinical monitoring was performed.

**Results:**

By focusing on the timing of different ICP patterns that were identified manually by clinicians, we detected significant tissue oxygen saturation (${\mathrm{StO}}_{2}$) changes (p=0.002) and blood flow index (BFI) variability (p=0.005) between regular and high-amplitude B-wave patterns. A blinded analysis looking for analogs of ICP patterns in BFI time traces achieved 90% sensitivity in identifying B-waves and 76% specificity in detecting the regular patterns.

**Conclusions:**

We revealed the presence of ${\mathrm{StO}}_{2}$ and BFI variations—detectable with optical techniques—during ICP B-waves in BEH children. Finally, the feasibility of detecting ICP B-waves in hemodynamic time traces obtained noninvasively was shown.

## 1 Introduction

Benign external hydrocephalus (BEH) is a condition usually diagnosed during the first year of life in infants presenting with macrocephaly or a rapidly increasing head circumference (HC). Neuroradiological findings show enlarged subarachnoid spaces—specifically at the frontotemporal lobes— and normal or moderately enlarged ventricles.^1^^,^^2^ BEH is commonly considered a self-limiting condition that does not require any treatment, but some children may present temporary or permanent psychomotor delays.^3^^–^^7^ Fine, gross motor and attentional skills have been identified as the most compromised developmental areas in infants with BEH.^7^^–^^10^ Additional complications, such as an increased risk of subdural hematoma and hypotonia, have also been reported.^4^^,^^11^^,^^12^

These findings complicate the management of BEH. In particular, there is still no consensus among clinicians about the effects of BEH on brain development and its optimal management. There is a general agreement that the attitude at diagnosis should be a wait and see approach, but there is also emerging evidence that some children require surgical treatment and the placement of a ventriculoperitoneal shunt.^13^ When in doubt, intracranial pressure (ICP) monitoring is useful in deciding which patients are good candidates for shunting. This is motivated by the observation that some BEH children present abnormalities in cerebrospinal fluid (CSF) dynamics that can be observed as quantitative and qualitative abnormalities in ICP recordings. These alterations may induce changes in cerebral oxygenation and blood flow, which, in turn, may lead to neurodevelopmental delays.^5^^,^^14^^,^^15^

ICP abnormalities that are observed do not necessarily manifest themselves as alterations of the mean ICP value; therefore, in these patients, the mean ICP is not enough for detecting abnormalities of CSF dynamics.^16^ There is more to ICP time traces. Of particular interest here are B-waves, which were first described by Lundberg as short repeating elevations of ICP, occurring at a frequency of 0.5 to 2 ICP cycles per minute and lasting at least 10 min^17^ with high (equal or above 10 mmHg) or low (below 10 mmHg) amplitude.^16^ The presence of B-waves is indicative of reduced intracranial compliance, and they appear mainly during the rapid eye movement (REM) sleep when there is an increase in cerebral blood flow (CBF) and brain metabolism.^18^^–^^21^ The alteration of cerebral autoregulation or reactivity due to reduced intracranial compliance could be pathological in such a scenario.

This observation was the main motivation for our study. We posit that the detection of B-waves is relevant to evaluating these infants, but continuous ICP monitoring is rarely prescribed to these populations due to its invasiveness and safety considerations. Therefore, there is a niche need to evaluate whether noninvasive, bed/cot-side monitoring of surrogates of ICP alterations could be utilized to detect B-waves. In this study, our working hypothesis was that the ICP alterations present in BEH could be detected using noninvasive, hybrid near-infrared spectroscopies. In particular, we employed near-infrared time resolved spectroscopy (TRS) and diffuse correlation spectroscopy (DCS). In brief, both techniques utilize near-infrared light with TRS deriving the microvascular, cortical concentration of oxy- and deoxy-hemoglobin (${\mathrm{HbO}}_{2}$ and Hb)^22^^,^^23^ and DCS obtaining an index proportional to microvascular, cortical CBF.^24^^,^^25^ These techniques have been shown to be a good tool to study the cerebral hemodynamics noninvasively at bedside in healthy and pathological conditions both in adult and pediatric populations. Near-infrared spectroscopy (NIRS) has been applied to the study of infant brain in healthy and pathological conditions;^26^^–^^30^ among others, it has been used to measure the changes in cerebral blood volume, cerebral tissue oxygenation (${\mathrm{StO}}_{2}$), and relative cerebral metabolic rate of oxygen.^31^^–^^33^ DCS has been validated for the assessment of CBF changes in adult and infant brains.^34^^–^^37^

Our aim was to conduct a proof-of-concept study using optical neuromonitoring to study cerebral oxygenation and blood flow in BEH children presenting ICP alterations. We sought to identify and quantify hemodynamic changes associated with these ICP alterations and to evaluate whether these alterations could be detected by optical monitoring alone.

## 2 Materials and Methods

The clinical leg of the study was carried out at the Pediatric Neurosurgery Unit of the Vall d’Hebron University Hospital (VHUH), Barcelona, Spain. The study was approved by the VHUH Ethical Committee (PR-ATR-402/2017) and was carried out in accordance with the Code of Ethics of the World Medical Association (declaration of Helsinki).^38^ The parents were asked for written informed consent before the inclusion. The study inclusion criteria were as follows: (1) children with a diagnosis of BEH and persistent neurodevelopment delay and/or clinical symptoms of increased ICP associated with macrocephaly or rapidly increasing HC during the first year of life and (2) a clinical indication for continuous ICP monitoring to establish a potential need for a CSF shunt.

### 2.1 Clinical Protocol

Most children with suspected BEH were referred to the Pediatric Neurosurgical Unit of the Neurosurgical Department at the VHUH by a pediatrician or a pediatric neurologist. A pediatric neurosurgeon conducted the first clinical evaluation, during which the clinical history of the child and the family was collected. BEH was defined as enlarged subarachnoid spaces in children with HC above the 97.5th percentile according to Spanish population, or rapidly increasing HC during the first year of life (at least crossing two percentiles of the normal values for the age), with normal ventricular size or mild ventriculomegaly. Moreover, the HC of the parents was measured, and they were classified as being macrocephalic according to the criteria described above for the children.^39^

**Fig. 1:**
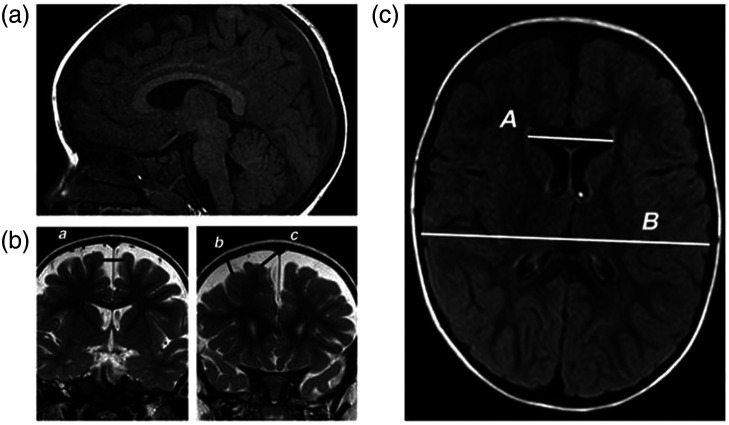
Example of a 33-month-old female born in a eutocic delivery (gestational age: 35 weeks, weight = 2020 g, height 45 cm, and HC = 33 cm), with an Apgar score (that is a standard neonatal health assessment score) of 6-9-9 at 1, 5, and 10 min of delivery. She was referred to a pediatric neurosurgeon for evaluation of hypotonia and enlargement of subarachnoid spaces. MRI showed the characteristic findings of benign enlargement of subarachnoid spaces in the frontal lobes [(a) sagittal T1-weighted MRI and (b) coronal T2-weighted MRI images): a, size of the interhemispheric fissure (12 mm); b, craniocortical width (8.4 mm); and c, sinocortical width (12.4 mm). (c) (axial T1-weighted MRI), the Evans’ Index (0.29) was calculated as the ratio between the maximum width of the frontal horns of the lateral ventricles (A=31  mm) and the maximum transverse inner diameter of the skull at the same axial slice (B=105  mm).

In each patient, the size of the extraventricular CSF compartment was measured along the frontal convexities at the coronal slices in a transfontanellar ultrasound study or magnetic resonance imaging (MRI) to calculate the following measures: the craniocortical width, the sinocortical width, and the width of the anterior part of the interhemispheric fissure (Fig. 1). The diagnosis of BEH required that at least one of the three measurements was >10  mm independent of sex.^6^^,^^40^ The ventricular volume—in transfontanellar ultrasound, computer tomography (CT) scan, or MRI—was estimated using the Evans’ index,^41^^,^^42^ calculated as the maximum width between the frontal horns of the lateral ventricles and the maximum transverse inner diameter of the skull at the same axial slice in the CT scan/MRI, or in the same coronal slice in the transfontanellar ultrasound. We introduce Fig. 1 to illustrate typical findings and the procedure.

The psychomotor development was evaluated by two trained evaluators (F. M. and L. G.) in all children. The goal was to evaluate all children using the third edition of the Bayley Scales of Infant and Toddler Development (Bayley-III).^43^ Those who were above the age threshold for the Bayley III were evaluated with the Wechsler Preschool and Primary Scale of Intelligence (WPPSI-IV). The presence of clinical symptoms was evaluated by an expert neurosurgeon (M. A. P.). For ICP monitoring, institutional practices were followed. At VHUH, continuous ICP monitoring in BEH is indicated when the child presents a persistent neurodevelopmental delay and/or clinical symptoms suggesting intracranial hypertension (irritability, frequent night waking, headache, and vomiting) associated with macrocephaly or rapidly increasing HC during the first year of life. Epidural ICP monitoring is performed for at least 72 h.

### 2.2 ICP Monitoring and Shunting Criteria

The ICP was measured through an epidural sensor (Neurodur-P^®^, Raumedic AG, Germany) placed into the frontal left epidural space. The sensor was inserted through a burr hole following the pupilar line and in front of the coronal suture. The ICP sensor was connected to an ICP monitor (MPR2 logO DATALOGGER, Raumedic AG, Germany). The ICP signal was sampled at 200 Hz and stored on a personal computer using a computer-based data acquisition and analysis system (PowerLab 4SP hardware and LabChart v8.1 software; ADInstruments, Ltd., Grove House, Hastings, UK).

A comment about this type of sensor should be made since in the literature an overestimation of the absolute ICP values when using epidural ICP sensors with respect to the parenchymal or ventricular ones has been reported in adults.^44^^,^^45^ In children, the epidural sensor is more reliable because dura mater is more easily detached from the internal table of the skull, thus reducing the differences in the absolute ICP values obtained in adults compared with other intracranial compartments. For the purposes of this study, the qualitative information obtained from the epidural sensors (frequency and amplitude of A- and B-waves) has been demonstrated to be equivalent to the quantitative data and valid.^44^ The ICP criteria for identifying abnormal CSF dynamics were described elsewhere.^46^ Here the presence of mean ICP>15  mmHg and/or the presence of A-waves (defined as ICP elevations at least 20 mmHg above the resting line, with abrupt onset and ending, and lasting between 5 and 20 min)^17^ and/or more than 20% of B-waves in the total duration of the nocturnal recording time were considered to be criteria for shunting following standard procedures of the hospital.

The ICP data were analyzed by an expert neurosurgeon (M. A. P.), and the different segments of the ICP recordings were categorized in one of the following profiles: (a) normal ICP profile, characterized by what we call a “regular pattern” (i.e., mean ICP<15  mmHg with a stable recording and without any pathological waves), (b) low-amplitude B-waves pattern (presence of B-waves with an amplitude<10  mmHg), (c) high-amplitude B-waves pattern (presence of B-waves with an amplitude≥10  mmHg), and (d) measurement artifacts.

### 2.3 Noninvasive Optical Monitoring

The optical monitoring was performed with a hybrid platform using both TRS and DCS combined in a single instrument and probe, similar to those in references.^47^^–^^51^ Briefly, TRS and DCS data were acquired at sampling rates of 1 and 2.5 Hz, respectively. The two techniques were synchronized together via a homemade software. The TRS hardware had two pulsed laser sources at 690 and 830 nm (PicoQuant GmbH, Germany) and two time-correlated single-photon counting cards (Becker&Hickl, GmbH, Germany). The DCS hardware had two continuous wave (CW) laser sources (CrystaLaser, USA) at 785 nm, eight avalanche photodiodes detectors (Excelitas, USA), and a hardware correlator (correlator.com, Germany). We employed two soft black probes, with fibers for the injection and detection of light being arranged by alternating DCS and TRS and shining light simultaneously. TRS employed multimode fibers for both injection [numerical aperture (NA) = 0.28] and detection, using bundles with a 5 mm of diameter (Fiberoptic Systems Inc., USA); instead, DCS light was conveyed by a multimode fiber (NA = 0.39) and collected by a bundle of four single-mode fibers (each with NA = 0.12, Fiberoptic Systems Inc., USA).

**Fig. 2:**
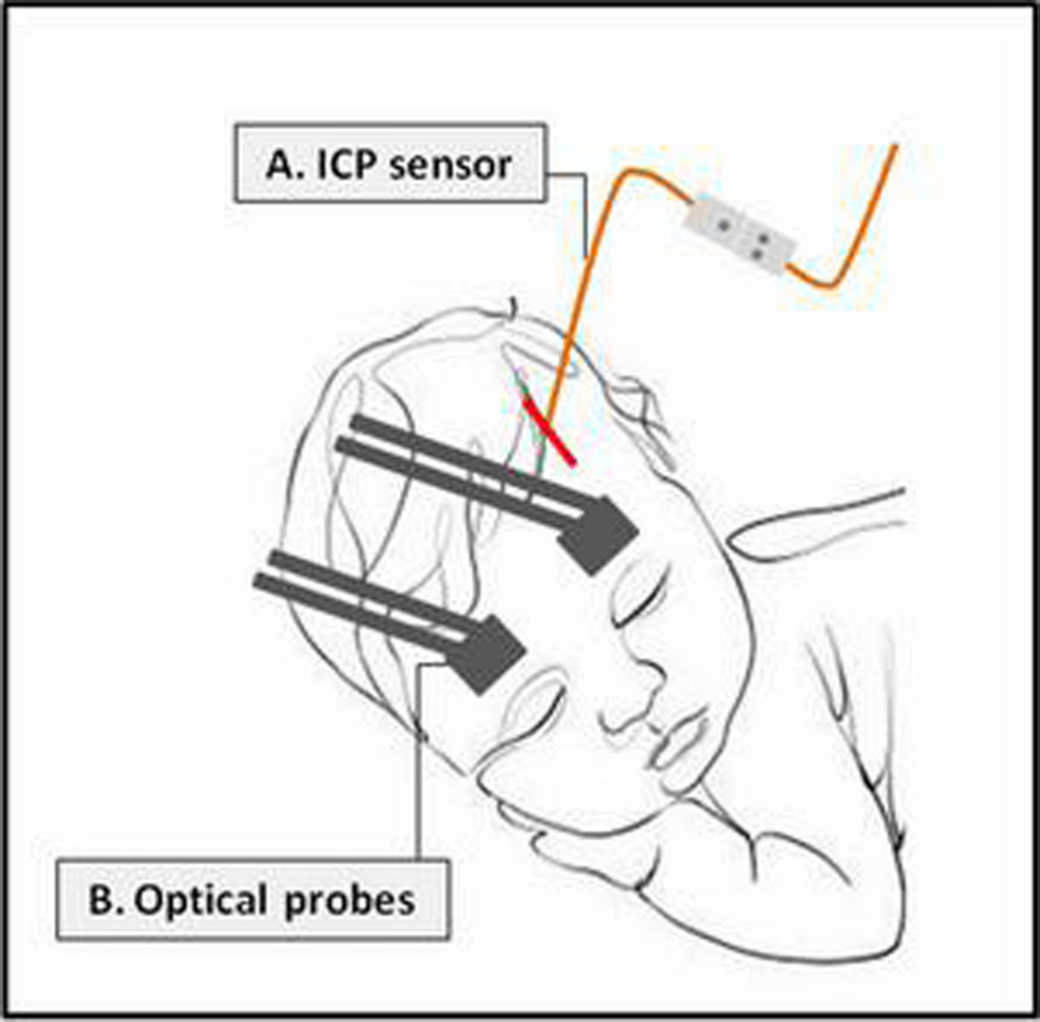
An illustration of the nocturnal intracranial (ICP) and optical monitoring. The ICP sensor was located in the frontal left epidural space (A), and the optical probes were placed on the forehead (B).

Initially, we used a source–detector separation (SDS) of 2.5 cm, but from subject five onward we used smaller probes that are more suitable for pediatric measurement with a SDS of 1.5 cm, which was previously validated.^25^^,^^31^^,^^34^^,^^52^^,^^53^ We also employed a smaller fiber holder patch (probe). We used the same fibers by placing them closer. The probes were placed on the child’s forehead just above the eyebrows to be able to monitor the frontal lobes and wrapped around the head with a skin compatible material (Fig. 2).

The synchronization between the optical and ICP measurements was realized through the LabChart software v7.0.3 (ADInstruments, New Zealand) and the data acquisition hardware PowerLab (ADInstruments, New Zealand). The ICP signal was sent from the monitor to the PowerLab, and the correlator sent a 10-Hz digital signal to PowerLab as a timing basis. The child’s movements or other potential artifacts were recorded in real time by the researchers (F. M., J. F., and S. T.) by inserting a digital mark both in the optical and the ICP recordings. After the measurement, the TRS data were processed, and ${\mathrm{HbO}}_{2}$ and Hb as well as total hemoglobin concentrations (THC) and ${\mathrm{StO}}_{2}$ were calculated as time traces.^12^ The DCS measurements also quantified the blood flow index (BFI) as being proportional to CBF as a time trace.^24^

### 2.4 Nocturnal Monitoring

Multimodal monitoring was carried out during night sleep, i.e., nocturnally, during two consecutive nights. The recording started after the child fell asleep as decided according to the parent’s experience to create a situation as comfortable as possible. Nocturnal monitoring is associated with various benefits. For example, nocturnal ICP recordings were shown to be more reliable in children than day monitoring,^54^ B-waves, which are of primary interest here, occur more frequently during the night, especially in REM sleep,^20^^,^^21^ and ICP monitoring is prone to motion artifacts that are minimized during sleep.

### 2.5 Statistical Methods

Summary descriptive statistics were obtained for each variable. The median, minimum (min), and maximum (max) values were used for continuous variables, and percentages and frequencies were used to summarize the categorical variables. The statistical analysis was built on the general hypothesis that optical techniques are able to detect cerebral hemodynamic variations occurring during ICP B-waves. To verify this hypothesis, two different analyses, described in the next section, were performed.

Data are presented using time traces and tables. The statistical analyses were performed using R software v3.6.2 and the integrated development environment R Studio v1.2.5042 (RStudio, Inc., Boston, Massachusetts, USA);^55^ the packages “lme4”^56^ and “multicomp”^57^ were used. The MATLAB software^58^ (version R2018b, MathWorks, USA) was used for fitting the data and representing time traces.

#### 2.5.1 Changes of cerebral hemodynamics during ICP B-waves

As a first step of our analysis, we hypothesized that the optical variables obtained noninvasively through a combined TRS-DCS platform can show significant changes in the presence of ICP alterations (specifically the B-waves). We also tested the hypothesis that the optical variables can show an increased variability (i.e., significant changes of the standard deviation) when B-waves occur. After the acquisition, the optical data measured during both nights from each subject were analyzed and were synchronized with the ICP recordings. We conducted a first analysis by building linear mixed effect (LME) models. The clinical and optical parameters, i.e., Hb, ${\mathrm{HbO}}_{2}$, THC, ${\mathrm{StO}}_{2}$, BFI, and ICP, were identified as outcome variables and the subject ID as a random effect. The presence or not of ICP waves and the different ICP patterns (regular, low-, and high-amplitude B-waves) were defined as fixed effects in two different models. A likelihood ratio (LHR) test was conducted to compare the built models to identify the best model, and residuals were checked. Specifically, the model defining the presence of B-waves and the one defining the pattern as fixed effect were separately compared with the null model. The Bayesian information criterion (BIC) was checked to confirm that we were choosing the model that better fits the data: the lower BIC represented the model better fitting the data. We opted not to test whether the combination of measured variables gave further improvements in identifying B-waves to avoid overusing the dataset. When a model including different types of ICP patterns (regular, low-, and high-amplitude B-waves) resulted in a statistically significant improvement as evaluated by the LHR analysis, a *post hoc* contrast analysis was performed through a general linear hypotheses (GLH) method. This test was designed to compare regular pattern and low-amplitude B-waves, regular pattern and high-amplitude B-waves, and low- and high-amplitude B-waves. An additional LHR analysis was performed by dividing the cohort into two subgroups according to the SDS used for the measurement.

#### 2.5.2 Effect of demographic and clinical variables on cerebral hemodynamics

We also investigated the influence of demographic and clinical parameters of our cohort on the variation of the cerebral hemodynamics parameters obtained through noninvasive optical monitoring. Such parameters include psychomotor delay, presence of symptoms, prematurity, gender, and macrocephaly. Associations of the cerebral hemodynamics variables (THC, ${\mathrm{StO}}_{2}$, and CBF) with the parameters were checked. We considered the mean values of THC, ${\mathrm{StO}}_{2}$, and CBF during the regular period that is a period of inactivity (normal parameters) independently from the sleep stage. To do so, a linear regression model was built.

Additional variables that could somehow influence the studied parameters were also analyzed. In fact, we hypothesized that the widespread range of ages and gestational ages of the children could affect the behavior of the measured parameters. We studied whether the probe’s distance from the brain could affect the optical signal. We assessed the influence of the HC because it is well known that extracerebral contamination increases as the upper layers get thicker. Finally, we tested the influence, as a fixed variable, of the probe type because two different probes with an SDS of 2.5 and 1.5 cm were utilized. Other additional variables (age in months or gestational age or HC) were tested to see if they contributed to the LME model by adding them to the pattern fixed effects. The interactions between these variables and the identified pattern (low- and high-amplitude B-waves) were also tested. For all of the mentioned variables, we performed multiple comparisons through LHR to test the null hypothesis of no difference between the mean and standard deviation of the null model and the model with pattern, between the model with pattern and the models with the additional variables, and the interaction between these two models. The comparison was considered significant when the second model improved the previous. A schematic diagram of the analysis and the R script used to carry it out are reported in the Supplementary Material.

Statistical significance was considered when p≤0.05. For multiple comparisons through LHR in the analysis of additional variables, a Bonferroni correction was applied and a corrected type error of 0.01 was established. The symbol << was used when the p value was very low (i.e., more than 0.001 decimals).

#### 2.5.3 Visual detection of ICP patterns in BFI time traces

The common practice for the evaluation of the ICP recording is the visual inspection of the time traces searching for B-waves. We observed a similarity between ICP and BFI time traces, so we hypothesized that an observer—blinded to the ICP data—can identify and distinguish the ICP patterns by looking at the BFI tracing of each subject. To verify the hypothesis, a blinded researcher (F. M.) carried out a visual detection of ICP patterns in BFI and marked regular and B-waves segments. We decided to not indicate the B-wave type (low and high amplitude) due to the relatively small sample size and excluded the periods with ICP artifacts from this analysis *a priori*.

We were interested in obtaining the sensitivity (i.e., our ability of recognizing the B-waves) and specificity (i.e., our ability of identifying the regular pattern) of our analysis. To calculate them, we compared the patterns identified by the blinded observer in BFI with the gold standard, which is the ICP patterns identified by the experienced neurosurgeon (M. A. P.). We defined the correctly identified B-waves as being true positive (TP) and the regular patterns as being true negative (TN), all from the noninvasive recording of BFI. Furthermore, regular patterns marked as B-waves were identified as being false positive (FP), and B-waves marked as regular patterns were a false negative (FN). The sensitivity was calculated as TP/(TP + FN), and the specificity was TN/(FP + TN).

## 3 Results

### 3.1 Clinical and Psychomotor Assessment

The recruitment lasted from November 2017 to June 2020 and included 12 children diagnosed with BEH that required continuous ICP monitoring. The data from one child were excluded because of poor optical signal quality. This subject was, in fact, awake, and the measurement was affected by movement artifacts. The final cohort included 11 children (5 girls) with a median age of 16 months (7 to 55 months).

The demographic and clinical data according to the inclusion criteria are summarized in Table 1. All children had a diagnosis of BEH. Macrocephaly was present in seven children (63.6%), and four (36.4%) presented a rapidly increasing HC during the first year of life. According to the age thresholds, the psychomotor development was evaluated in ten children using the Bayley-III scales^43^ and in one child using the WPPSI-IV. In nine children (81.8%), a persistent neurodevelopmental delay was detected. All patients presented clinical symptoms of increased ICP. Hypotonia was present in eight (72.7%), irritability in two (18%), headache in two (18%), and night waking in two (18%) children. Additional parameters include prematurity (five children), a positive family history for macrocephaly (one child), and hydrocephalus (one child), with associated problems (six children). All children needed ICP monitoring to evaluate if the placement of a ventriculoperitoneal shunt was necessary.

**Table 1:** Demographic and clinical data of the BEH patients (n=11).

Sex: boys/girls	6 (54.5%)/5 (45.4%)
Age in months (median, min, and max)	16 [7 to 55]
Gestational age	
Very preterm (28 to 31 week)	1 (9%)
Moderate preterm (32 to 33) week)	2 (18%)
Late preterm (34 to 37 week)	2 (18%)
Full term birth (38 to 42 week)	6 (54.5%)
HC	
Macrocephaly (HC > 97.5th)	7 (63.6%)
Rapidly increasing HC	4 (36.4%)
Positive family history	
Macrocephaly	1 (9%)
Hydrocephalus	1 (9%)
Associated problems	
Achondroplasia	2 (18%)
Genetic syndrome	2 (18%)
Subdural hematoma	1 (9%)
Chiari malformation type 1	1 (9%)
Persistent neurodevelopmental delay	9 (82%)
Cognitive area (Bayley-III)	2
Language area (Bayley-III)	5
Motor area (Bayley-III)	8
Language area (WPPSI-IV)	1
Planning (WPPSI-IV)	1
Clinical symptoms	
Hypotonia	8 (72.7%)
Irritability	2 (18%)
Headache	2 (18%)
Night waking	2 (18%)

Results are expressed as N (%). GA: gestational age; HC, head circumference; and WPPSI, Wechsler preschool and primary scale of intelligence.

### 3.2 Epidural ICP Monitoring, Shunt Placement, and Follow-Up

As planned, continuous ICP monitoring was carried out in all children. During the simultaneous noninvasive optical study and epidural ICP monitoring, the median ICP value was 18.5 mmHg (min: 13 mmHg and max: 26.1 mmHg). Of the total recording time, 114 periods of low-amplitude B-waves and 84 of high-amplitude B-waves were identified by the expert neurosurgeon, giving a total of 198 ICP periods with B-waves. The median percentage of B-waves was 61% (min: 47 and max: 97) of the total duration of the recordings. Of these, low- and high-amplitude B-waves were divided approximately evenly (∼50% to 50%). Only one patient presented plateau waves, which led us to discard plateau waves (A-waves) from the analysis. The ICP monitoring and clinical practice led to the placement of a ventriculoperitoneal shunt in all patients. A clinical and psychomotor follow-up was performed at 6 and 12 months after the surgery.

### 3.3 Optical Monitoring

The optical data acquired from the same hemisphere in which the epidural ICP sensor was implanted (frontal region of the left hemisphere) were used for the analysis because they showed a slightly better signal quality upon a qualitative evaluation. As planned, the optical measurement was performed during two consecutive nights (median time per night = 6 h, min: 2, and max: 7) per subject. DCS data were acquired for the whole cohort of 11 subjects. TRS data were acquired for nine subjects because of technical issues during the measurements of the remaining two subjects. From the initial 198 segments identified as B-waves, 32 were excluded because they comprised B-waves already started at the beginning and/or still ongoing at the end of the optical measurement. Furthermore, the so-called plateau waves were also identified, but because their appearance is quite rare in these children, they were not included in the analysis. Therefore, a total of 166 periods with B-waves and 60 regular segments that were detected in the ICP recordings were compared with the noninvasive optical data through an LHR analysis.

#### 3.3.1 Changes of cerebral hemodynamics during ICP B-waves

The LHR analysis was applied to two models (one indicating the presence or not of B-waves and one including different patterns (regular, low-, and high-amplitude B-waves)). Both models were separately compared with the null one. ICP and ${\mathrm{StO}}_{2}$ showed significant changes during B-waves (p≪0.001 and p=0.01, respectively). Specifically, the presence of different patterns showed a significant increase of ICP and ${\mathrm{StO}}_{2}$ with respect to the null model (p≪0.001 and p=0.001, respectively). Moreover, the presence of B-waves revealed a significant increased variability of ICP and BFI with respect to the null model (p≪0.001 and p=0.003, respectively). A significant variability of ICP and BFI was also detected during different patterns with respect to the null model (p≪0.001 and p=0.01, respectively). The analysis was performed including the whole cohort (eleven subjects for ICP and BFI and nine subjects for ${\mathrm{StO}}_{2}$ and THC). Detailed results are presented in Table 2. A further analysis was performed with a subcohort having both TRS and DCS measurements including nine subjects. There was no difference in significance between the two analyses. To confirm that we could use the subjects measured with different SDSs as a group, we also conducted the LHR analysis separately for the subjects measured with a long SDS (n=4) and the ones measured with a short SDS (n=7), thus finding no statistical difference between the two analyses. For the first group (long SDS), ICP and ${\mathrm{StO}}_{2}$ showed significant changes during B-waves (p≪0.001 and p=0.003, respectively). A significant change of THC during B-waves was also detected for this group (p=0.03). The second group (short SDS) showed significant changes of ICP during B-waves (p≪0.001) and of ICP and ${\mathrm{StO}}_{2}$ during different patterns (p≪0.001 and p=0.01, respectively). Moreover, the presence of B-waves revealed a significantly increased variability of ICP and BFI with respect to the null model both in the first (p≪0.001 and p=0.04, respectively) and second group (p≪0.001 and p=0.01, respectively).

**Table 2:** Optical variables characterization during different ICP patterns.

**Variable**	**Mean [min to max]**		
	**Regular**	**High B waves**	**Low B waves**
ICP ** (mmHg)	15.6 [8.7 to 27.4]	20.05 [4.8 to 31.5]	18.1 [9.8 to 33.1]
THC (μM)	70 [40 to 100]	70 [40 to 100]	70 [40 to 100]
${\mathrm{StO}}_{2}$* (%)	60.5 [49.7 to 76.1]	62 [51.4 to 73.6]	60.3 [51.1 to 74.5]
BFI (${\mathrm{cm}}^{2}/s$)	$4.2\times 10^{-8}$ [$8.4\times 10^{-9}$ to $1.1\times 10^{-7}$]	$3.7\times 10^{-8}$ [$9.5\times 10^{-9}$ to $1.3\times 10^{-7}$]	$2.9\times 10^{-8}$ [$1.04\times 10^{-8}$ to $1.1\times 10^{-7}$]
**Variable**	**Standard deviation [min to max]**		
ICP^^ (mmHg)	1.7 [0.4 to 6.8]	4.7 [1.2 to 13.2]	3.1 [0.5 to 8.2]
THC [μM]	6 [3 to 10]	5 [2 to 10]	4 [2 to 10]
${\mathrm{StO}}_{2}$ (%)	5.6 [2.5 to 9.2]	4.8 [2.03 to 7.7]	5.1 [2.3 to 9.5]
BFI^ (${\mathrm{cm}}^{2}/s$)	$6.3\times 10^{-9}$ [$3.4\times 10^{-10}$ to $2.5\times 10^{-8}$]	$6.5\times 10^{-9}$ [$8.9\times 10^{-10}$ to $2.6\times 10^{-8}$]	$5\times 10^{-9}$ [$5\times 10^{-10}$ to $2.6\times 10^{-8}$]

Note: mean: *p<0.05; **p<0.001.

standard deviation: ^p<0.05; ^^p<0.001.

A *post hoc* analysis through GLH was applied to study which patterns were causing the significant changes reported in the previous analysis. Specifically, ICP showed a significant increase with respect to regular pattern during high-amplitude B-waves (p≪0.001) and low-amplitude B-waves (p≪0.001). Furthermore, high-amplitude B-waves showed a higher increase in ICP compared with low-amplitude B-waves (p≪0.001). ${\mathrm{StO}}_{2}$ also showed a significant increase compared with the regular pattern during high-amplitude B-waves (p<0.001), and they were also higher for high-amplitude compared with low-amplitude B-waves (p=0.01).

The same analysis was applied to study variability as described above. ICP presented a significant variability during both high-amplitude (p≪0.001) and low-amplitude (p≪0.001) B-waves with respect to the regular pattern. The ICP variability was also higher during high-amplitude B-waves with respect to the low-amplitude B-waves (p≪0.001). BFI showed higher variability also for both high-amplitude (p=0.01) and low-amplitude B-waves (p=0.02) but not between two types of B-waves.

#### 3.3.2 Effects of demographic and clinical variables on cerebral hemodynamics

The demographic and clinical parameters did not show any significant effect on the measured variables. Specifically, THC, ${\mathrm{StO}}_{2}$, and CBF were not associated with the presence of psychomotor delay, neither with the presence of symptoms nor with prematurity, gender, or macrocephaly (p<0.05). Similarly, the analysis of the associations between the optical parameters and additional variables (such as age in months, GA, HC, and probe type) revealed no significant effect on the parameters measured through optics (p<0.05).

#### 3.3.3 Visual detection of ICP patterns in BFI time traces

**Fig. 3:**
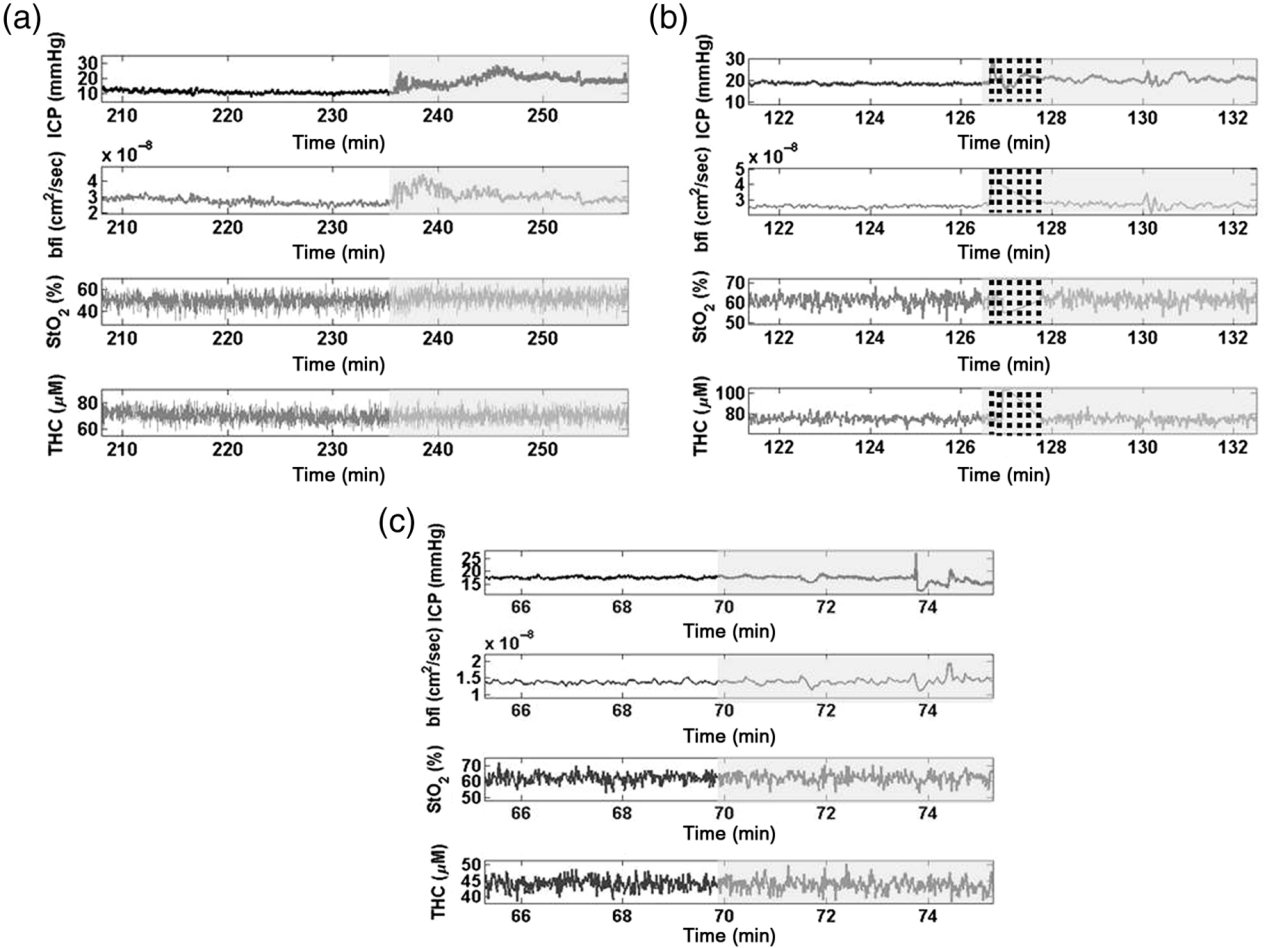
(a)–(c) Three examples of data acquired through the simultaneous ICP and optical monitoring of three different subjects, respectively, 7-, 12- and 31-month-old, are shown. Regular pattern (white area) and B-waves (gray area) are marked in the measured variables. In subject (a), the B-waves were correctly distinguished from the regular pattern at the blind visual detection in BFI; in subject (b), they were not identified; and in patient (c), they were identified even though the difference from the regular pattern was subtle. Movement artifacts are represented through dashed lines. BFI, blood flow index; ICP, intracranial pressure; ${\mathrm{StO}}_{2}$, tissue oxygen saturation; and THC, total hemoglobin concentration.

**Fig. 4:**
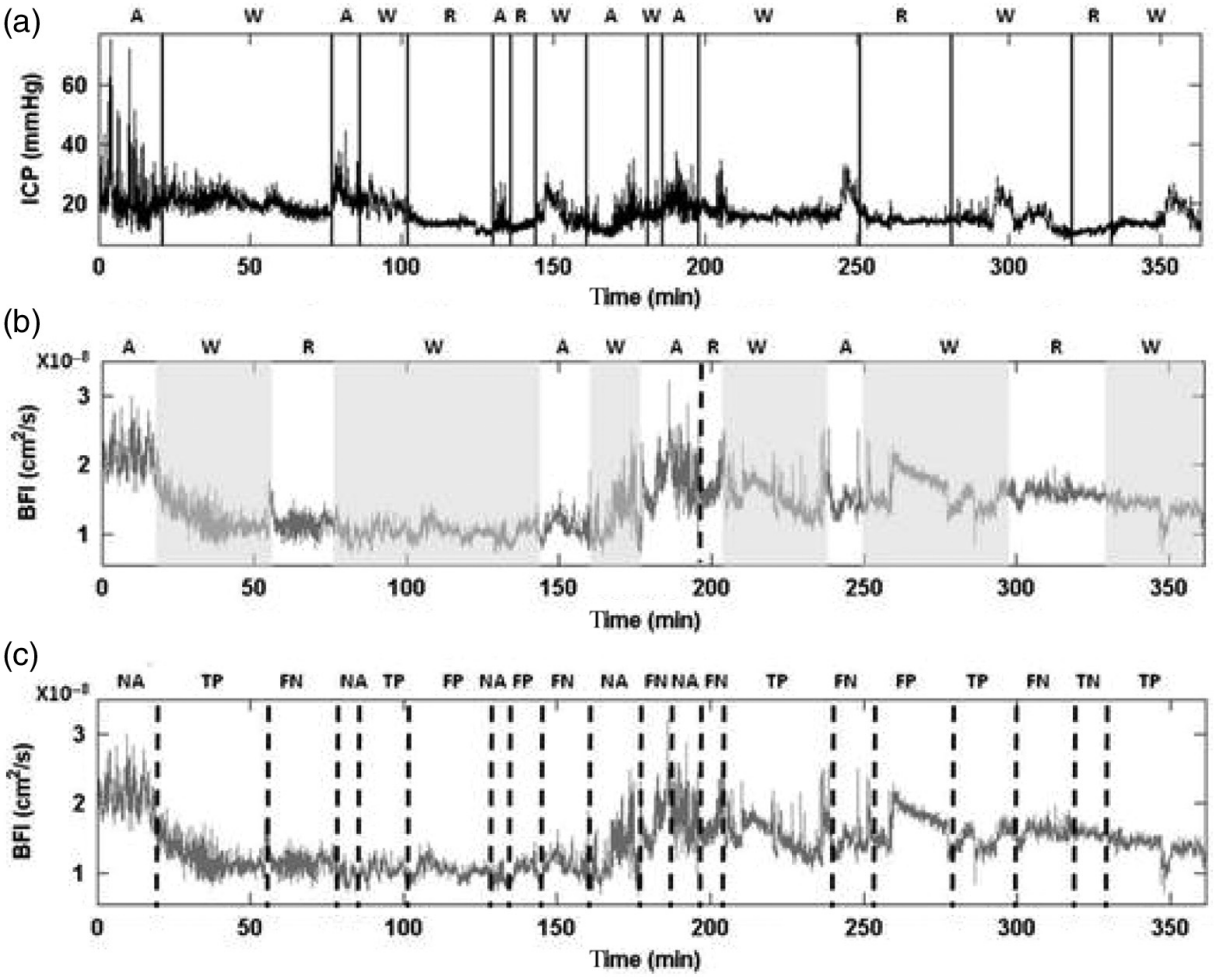
Six-h and 3-min measurement of an 11-month-old child are shown. (a) The ICP analysis done by the neurosurgeon is shown. (b) The blinded visual detection and (c) the comparison to calculate sensitivity and specificity are presented. TP indicates B-waves correctly identified as B-waves, FP regular pattern identified as B-waves, TN regular pattern correctly identified as regular, and FN B-waves identified as regular pattern. In this example, six B-waves over seven and one regular pattern over four were correctly identified. In BFI, regular patterns/artifacts are represented as white areas and B-waves as gray areas. In (c), the outcome of the analysis is shown. It represents the correspondence between the patterns identified by (a) the neurosurgeon and by (b) the blinded researcher. A, artifact; BFI, blood flow index; FN, false negative; FP, false positive; ICP, intracranial pressure; NA, not applicable; R, regular; TN, true negative; TP, true positive; and W, waves.

As stated in the methods, for this analysis, we did not distinguished between high- or low-amplitude B-waves, and wherever the neurosurgeon identified a high-amplitude B-waves followed by a low-amplitude B-wave, for the sensitivity calculation, it was counted as a single B-wave. The total number of B-waves periods was initially 167, but the waves counted for the sensitivity analysis were, therefore, 87 and 60 regular segments in the ICP recordings. Figures 3 and 4 show some examples of the different patterns detected in the simultaneous invasive ICP and optical recordings. In Fig. 3, the pattern analysis done by the blind observer (F. M.) for three different subjects is shown. This figure illustrates different scenarios of the blinded analysis: when the patterns are correctly identified in BFI, when they are not identified, and when the patterns are caught correctly by the blind observer even though the distinction between regular pattern and B-waves is subtle. In Fig. 4, we present an example of one night measurement with marked ICP abnormalities detected by the neurosurgeon and the BFI data analysis made by the blind observer. Figure 4(c) shows the identified or not identified patterns in BFI. All of the patterns were used to calculate the sensitivity and specificity. Finally, we identified 78 B-waves segments in BFI time traces over 87 and 43 regular segments in BFI over 60 in the ICP recordings. Considering all data, a sensitivity of 90% [confidence interval (CI) 95% 82 to 94] in the detection of B-wave pattern and a specificity of 76% (CI 95% 63 to 85) in the detection of regular patterns in the optical data were obtained.

## 4 Discussion

We used an innovative and noninvasive optical technique to monitor children affected by BEH during nocturnal, invasive ICP monitoring. In this study, we were able to detect quantitative changes in cerebral hemodynamic parameters obtained through optical techniques during the appearance of the so-called B-waves. Specifically, when the ICP recording revealed the presence of B-waves, we detected a significant increase in ${\mathrm{StO}}_{2}$ (p=0.01). We also detected a significant increase of standard deviation of BFI in the presence of B-waves (p=0.003). Because the analysis of the ICP tracing is made by searching for B-waves manually by eye, we carried out an analogous analysis in the BFI data. We reported a good sensitivity (90%, CI 95% 82 to 94) and specificity (76%, CI 95% 63 to 85) to detect and distinguish B-waves. These findings overall motivate us to further study nocturnal optical monitoring as a means to characterize the presence and the potential deleterious effects of ICP waves in this population without the need for invasive ICP monitoring. This could complement the clinical practice and knowledge.

In a previous study,^59^ our group proved the feasibility of a method to estimate ICP from pulsatile, microvascular CBF data through a recurrent neural network in a population of six infants with BEH and six adults with traumatic brain injury. We found a high correlation (R=0.95) and a mean difference of +82% in the Bland–Altman analysis between the invasive and the predicted ICP for the BEH cohort. For the adult cohort, a good correlation (R=0.96) and a bias between the two methods of +0.69% were detected.

### 4.1 Noninvasive Optical Techniques to Study Pathological Alterations in ICP

The ICP monitoring reveals important information to address the management of children with BEH, but it is still an invasive technique. The risks associated with the insertion of a sensor into the cranium include hemorrhage and infection.^60^ There is also a risk of device failure during insertion or the sensor being accidentally explanted by the patient.^60^ In our series, none of the children presented any complications derived from the placement of the ICP sensor. However, the implementation of noninvasive techniques to study conditions of increased ICP in the pediatric population is desirable.

We looked at a noninvasive way to obtain information about cerebral hemodynamics in BEH children using optical techniques contemporary to the standard ICP monitoring. A deeper knowledge about the pathophysiology of this syndrome could open the path to a future in which invasive techniques can be replaced by, or at least used in combination with, noninvasive methods in the pediatrics field. We used a noninvasive and cot-side device that combines TRS, for calculation of hemoglobin contents and oxygenation with newly developed DCS, for calculation of regional perfusion. The feasibility of such techniques for noninvasive continuous bedside CBF and oxygenation monitoring in the pediatric population was demonstrated.^33^^,^^34^^,^^61^ As stated in the methods section, for ergonomic reasons, from subject 5, we decided to improve the optical setup by adapting it to the age (implying different anatomy and forehead dimensions than adults) of our population. In other words, we switched to a short SDS because it has been previously validated.^25^^,^^31^^,^^34^^,^^52^^,^^53^ Moreover, in babies of this age, the skull thickness is quite small, allowing for good light penetration with no substantial difference between large and short SDSs.^56^^,^^57^ We also proved that the probe type had no influence on the results by testing it as an additional variable to the pattern fixed effects and by performing the LHR analysis with two separate groups (subjects measured with long and short SDS). This could, in the future, be further analyzed by detailed simulations. The decision of switching from a long to a short SDS was also made considering the objective of the study. In fact, our main objective was to verify if the presence of ICP B-waves could cause changes (even small ones) in optical parameters revealing specific patterns without necessarily having a common amplitude. To reach this goal, a high signal-to-noise-ratio and precision were crucial. Therefore, we applied two techniques, i.e., TRS and DSC, with demonstrated high sensitivity to the brain.^23^^,^^62^^,^^63^ On the one hand, TRS is considered the NIRS modality that allows for retrieving the optical properties (absorption and scattering) of the tissue and thus obtaining the absolute concentration of hemoglobin and tissue saturation. Moreover, depth sensitivity is reached due to the ability of detecting different time gates that represent the arrival time of the photons, thus allowing for differentiation between early (more sensitive to superficial layers) and late (more sensitive to deep layers) gates.^27^ Depth sensitivity is reached due to the ability of detecting the arrival times of the photons. On the other hand, DCS has a higher sensitivity to the brain and less contamination from scalp and skull compared with CW-NIRS due to the strong differential in the number of moving scatterers (red blood cells) in the upper layers (scalp and skull) versus the lower layers (brain) because DCS is preferentially sensitive to the moving scatterers. Specifically, Selb et al.^63^ demonstrated, through a Monte Carlo simulation on a head model, a relative brain-scalp sensitivity three times higher in DCS compared with that of CW-NIRS.

Previous studies have looked at the presence of slow oscillations, such as B-waves, in other signals obtained noninvasively. Spiegelberg et al.,^19^ in their review, described the attempts of detecting measurable parameters that can show oscillations in the same frequency range of ICP B-waves, calling them “B-wave surrogates.” The authors defined “B-wave surrogates as oscillations of signals associated with but different from ICP within the same frequency range as proper B-waves.” The frequency of B-waves was originally defined as 0.5 to 2 cycles per minute and recently redefined with an extended range of 0.33 to 3 cycles per minute. In this frequency range, surrogates were found in transcranial Doppler (TCD) and NIRS signals. The oscillations detected in TCD coincide with fluctuations of the blood flow velocity that happen in phase with ICP changes and can also occur in healthy subjects. By applying a hybrid optical technique, we were able to detect changes of cerebral hemodynamic parameters occurring in the B-waves frequency range. In fact, we have acquired the TRS data at a sampling rate of 1 Hz and a DCS of 2.5 Hz, thus being able to catch signals in the range of 60 to 150 cycles per minute.

Fluctuations have also been identified as possible markers of shunt responsiveness in hydrocephalus patients. Droste et al.^64^ referred to the presence of equivalents of B-waves (BWEs) in the TCD overnight monitoring of 10 healthy adults and in 11 patients with suspected normal pressure hydrocephalus (NPH). In the NPH subjects, these oscillations happened simultaneously with the ICP B-waves. The association of BWEs with B-waves in patients with NPH who were not improving after shunting has been demonstrated.^65^^,^^66^ Moreover, rhythmic oscillations of ICP associated with fluctuations in the TCD signal have been detected during sleep, and their variations in accordance with the sleep stage have been demonstrated.^66^ Specifically, there is an increase of BWEs during the REM phase. In our study, even though we performed a nocturnal monitoring and observed changes of the measured parameters in the presence of B-waves, we could not specify in which sleep stage they were occurring and we could not prove if their appearance was related to a specific sleep stage. The cited studies are in accordance with our results because they confirmed the presence of CBF velocity oscillations in the major arteries (in case of TCD) or microvascular hemodynamics (in case of NIRS) during ICP changes and revealed the importance of characterizing the B-wave surrogates in healthy subjects and in patients not intended to undergo invasive ICP monitoring.

TCD is an accepted clinical modality, but it has limitations. In many subjects, it is not possible to find an appropriate “bone window” to use TCD.^67^ TCD is also very sensitive to motion and probe placement and is often operator dependent.^68^^,^^69^ On the other hand, DCS is not a direct surrogate of TCD because DCS measures local changes in microvascular blood flow. This is both an advantage and a complication. The ability to look at local changes could allow specific regions to be targeted to understand the potentially deleterious effects of ICP waves. The complication arises because, in the presence of abnormalities or due to the thick skull/scalp, DCS signals may not reflect the cortical signals, i.e., they can be contaminated by extra cerebral signals. These complications are not so prominent in pediatric populations due to the smaller scalp to brain distance and appropriate regions being selected based on radiology images. Our results indicate that DCS is usable in this population. The recent emergence of commercial DCS systems (HemoPhotonics S.L., Spain and ISS Inc., USA) and various research projects carrying them to medical device approvals stage funded by the European Commission and the National Institutes of Health demonstrate that, in the near future, DCS could provide simplified, relatively low-cost, noninvasive instrumentation that is not operator-dependent.

Slow oscillations during ICP B-waves were also found in NIRS parameters, suggesting that this technique may be used as a noninvasive marker of ICP slow waves. Several attempts of characterization were made in both healthy and pathological conditions. Weerakkody et al.^70^ described a synchronization between slow ICP B-waves and Hb obtained through NIRS during controlled elevations of ICP (infusion test) performed in 19 patients with a history of CSF dynamic disorders. The mean frequency of slow waves was 1.32 (0.28) cycles per minute, with a range of 0.75 to 1.98 per minute. In this slow wave bandwidth, the presence of strong and regular slow waves of ICP coincided with waves of the same periodicity in Hb or ${\mathrm{HbO}}_{2}$. They observed high coherence between NIRS variables and ICP (>0.7) in a frequency range consistent with the slow ICP waves described by Weerakkody et al.^70^ Weerakkody et al.^71^ described the changes in ICP and the mutual character of cyclic fluctuations in Hb and ${\mathrm{HbO}}_{2}$ recorded through NIRS. They stated that slow ICP waves are accompanied by synchronous changes in Hb and ${\mathrm{HbO}}_{2}$ in phase with each other. The authors proved that slow fluctuations in NIRS variables appear during ICP slow waves. These studies are based on CW NIRS systems with limitations such as the impossibility of calculating absolute ${\mathrm{HbO}}_{2}$ and Hb values or their sensitiveness to motion artifacts with respect to time domain NIRS used in our work that are already known.

The presence of slow oscillations in Hb and ${\mathrm{HbO}}_{2}$ has also been detected in pathological conditions, such as severe head injury and subarachnoid hemorrhage.^72^ Cheng et al. detected oscillations of ${\mathrm{HbO}}_{2}$ in a frequency range compatible with B-waves in nine patients with a Glasgow coma scale<8. This implies that NIRS is able to detect such variations and could be used in situations of increased ICP. In contrast, we did not observe any significant changes in Hb or ${\mathrm{HbO}}_{2}$, but we detected a significant increase of ${\mathrm{StO}}_{2}$ during B waves (p=0.01). Moreover, we proved an increased variability of BFI during B-waves (p=0.003). Working with clinicians who were able to analyze the ICP by eye and distinguish between different patterns allowed us to study the effects of ICP patterns on the measured variables (THC, ${\mathrm{StO}}_{2}$, and BFI), achieving innovative information. Specifically, ${\mathrm{StO}}_{2}$ revealed a significant increase during high-amplitude B-waves with respect to the regular pattern (p<0.001) and during low-amplitude compared with high-amplitude B-waves (p=0.01); BFI showed a significant variability between regular pattern and high-amplitude B-waves (p=0.01) and between regular pattern and low-amplitude B-waves (p=0.02).

Attempts at identifying ICP variations noninvasively have also been made in the pediatric populations. Urlesberger et al.^73^ observed cyclic fluctuations of Hb and ${\mathrm{HbO}}_{2}$ in the frequency range of 3 to 6 cycles per minute in 58 healthy full-term infants. By looking at the amplitude of the fluctuations, they concluded that such fluctuations were in the normal ranges for parameters fluctuations in long-term NIRS tracings. Livera et al.^74^ investigated the presence of oscillations in the NIRS signal in the frequency range from 3 to 5 cycles per minute in preterm infants reporting cyclic fluctuations in THC. In these studies, the origin of such fluctuations remains unclear, and it is not related to a condition of pathological ICP. We were able to measure a population presenting ICP pathological B-waves and to characterize our signals during such oscillations.

The innovative approach in our contribution with respect to previous studies is given by the visual detection analysis performed by searching for analogs of ICP in BFI tracing. We obtained a good sensitivity (90%, CI 95% 82 to 94) in identifying analogs of ICP B-waves in BFI tracing. We were also able to detect regular ICP patterns, thus achieving a good specificity (76%, CI 95% 63 to 85). The visual analysis of noninvasive parameters variations in the presence of ICP B-waves could be studied in a larger cohort to confirm these findings and introduce optical techniques in addition to invasive monitoring. Such an advance is desirable, especially for the pediatric population and clearly in a syndrome such as BEH for which there is still confusion about its management. Given the fact that the prevailing approach among clinicians is conservative because the syndrome is considered to resolve spontaneously with age,^3^^,^^75^ it becomes fundamental to retrieve more information about cerebral hemodynamics than merely the ICP. Intracranial hypertension, in fact, could lead to permanent but potentially avoidable delays in these children.^6^^,^^8^^,^^9^ In our cohort, a pathological ICP and the need for a ventriculoperitoneal shunt was confirmed, thus supporting our hypothesis; we recorded a mean ICP of 18.5 mmHg (IQR 5.5, min: 13, and max: 26.1) and a median percentage of total B-waves of 61% (min: 47.3 and max: 96.6). The visual inspection revealed the presence of 114 ICP recording segments of low-amplitude B-waves, 84 of high-amplitude, and 3 plateau waves. All children included in the cohort were shunted.

Our results confirm that optical techniques can be used to monitor a pediatric cohort such as BEH children in a convenient way for the patients. First, they are safe and noninvasive, so there is no need for a surgical procedure. Second, the monitoring can be performed at bedside, continuously, and while the child is sleeping, thus not obliging him to not move during the daytime. The measurement can be adapted to the patient’s needs in terms of protocol and materials. Moreover, using a hybrid TRS-DCS device in combination with the standard ICP monitoring, additional information about cerebral hemodynamics in a condition of increased ICP and in the presence of ICP B-waves could be obtained.

### 4.2 Study Limitation

The population is rather small, and all of the subjects have shown ICP waves with very few artifactual data that were noted to exclude the affected segment. In the future, a large and more heterogeneous population could be studied, including children with and without invasive ICP monitoring.

The sensitivity and specificity of the optical data to identify B-waves were assessed by a single observer who was deeply involved in the study but was blinded to the ICP traces. We did not evaluate interobserver variability, and we did not employ independent observers. This needs to be validated on a larger scale. Even though the visual analysis of optical data is complementary to the ICP recordings analysis and did not drive any clinical decision, it could provide additional information.

DCS is a relatively new technique, and artifacts that may present themselves as ICP waves cannot be fully ruled out. Our (and others’) experience from NIRS suggests that powerful artifact identification and removal methods can be employed successfully, and as the field progresses, we expect to employ them.

Our methodology did not allow us to relate these findings to the developmental status of the children, and we did not include a long-term follow-up in this particular study. Although, some children will undergo such procedures.

## 5 Conclusions

We have demonstrated the feasibility of nocturnal optical monitoring in a BEH population using a hybrid near-infrared spectroscopic device. We collected innovative information about cerebral hemodynamic changes during ICP B-waves. Specifically, we found a significant increase of ${\mathrm{StO}}_{2}$ from regular to high-amplitude B-waves pattern and a significant variability of BFI during high-amplitude B-waves. In children, the visual detection of pathological patterns in ICP recording is considered relevant to drive the clinical management. We achieved good sensitivity and specificity in identifying B-waves and regular patterns in BFI time traces. To the best of our knowledge, this study is the first to assess the behavior of cerebral hemodynamic variables obtained noninvasively in a BEH cohort. The introduction of a noninvasive method could complement the gold standard ICP monitoring used in clinics and give additional and precious information about cerebral hemodynamics in this population.

## Code, Data, and Material Availability

The data that support the findings of this study are available from the corresponding author upon reasonable request.
