# Erratum: Transcranial optical monitoring for detecting intracranial pressure alterations in children with benign external hydrocephalus: a proof-of-concept study

**DOI:** 10.1117/1.NPh.10.4.049801

**Published:** 2023-12-13

**Authors:** Federica Maruccia, Susanna Tagliabue, Jonas B. Fischer, Michał Kacprzak, Santi Pérez-Hoyos, Katiuska Rosas, Ignacio Delgado Álvarez, Juan Sahuquillo, Turgut Durduran, Maria A. Poca

**Affiliations:** a Vall d’Hebron Barcelona Hospital Campus, Vall d’Hebron Research Institute, Neurotraumatology and Neurosurgery Research Unit, Barcelona, Spain; b ICFO-Insitut de Ciències Fotòniques, The Barcelona Institute of Science and Technology, Barcelona, Spain; c HemoPhotonics S.L., Barcelona, Spain; d Nalecz Institute of Biocybernetics and Biomedical Engineering, Warsaw, Poland; e Vall d’Hebron Research Institute, Statistics and Bioinformatics Unit, Barcelona, Spain; f Vall d’Hebron Hospital Universitari, Vall d’Hebron Barcelona Hospital Campus, Department of Neurosurgery and Pediatric Neurosurgery Unit, Barcelona, Spain; g Vall d’Hebron Hospital Universitari, Vall d’Hebron Barcelona Hospital Campus, Department of Pediatric Neuroradiology, Barcelona, Spain; h Universitat Autònoma de Barcelona, Barcelona, Spain; i Institució Catalana de Recerca i Estudis Avançats, Barcelona, Spain

## Abstract

The erratum corrects errors in Fig. 3 and Table 2 of the original published article.

This article [*Neurophotonics* **9**(4), 045005 (2022) doi: 10.1117/1.NPh.9.4.045005] was originally published on 17 November 2022 with two errors that do not impact the reported results:

1. In Figure 3, the StO2 and BFI curve were reversed.
2. In Table 2, there was a conversion error in THC values reported in micromolar.

The errors were corrected with updates to Fig. 3 and Table 2 on 9 December 2023.

