# Supplementary material for: Transcranial optical monitoring for detecting intracranial pressure alterations in children with benign external hydrocephalus: a proof-of-concept study
Source: Neurophotonics. 2022 Nov 17;9(4):045005. doi: 10.1117/1.NPh.9.4.045005 (PMC9670160; doi:10.1117/1.NPh.9.4.045005)
Supplement: Supplementary file 1 [file NPh_009_045005_SD001.pdf]

1

We report a schematic diagram of the analysis (Figure S1) and R script used to conduct the statistical analysis.

Supplemental Figure S1.

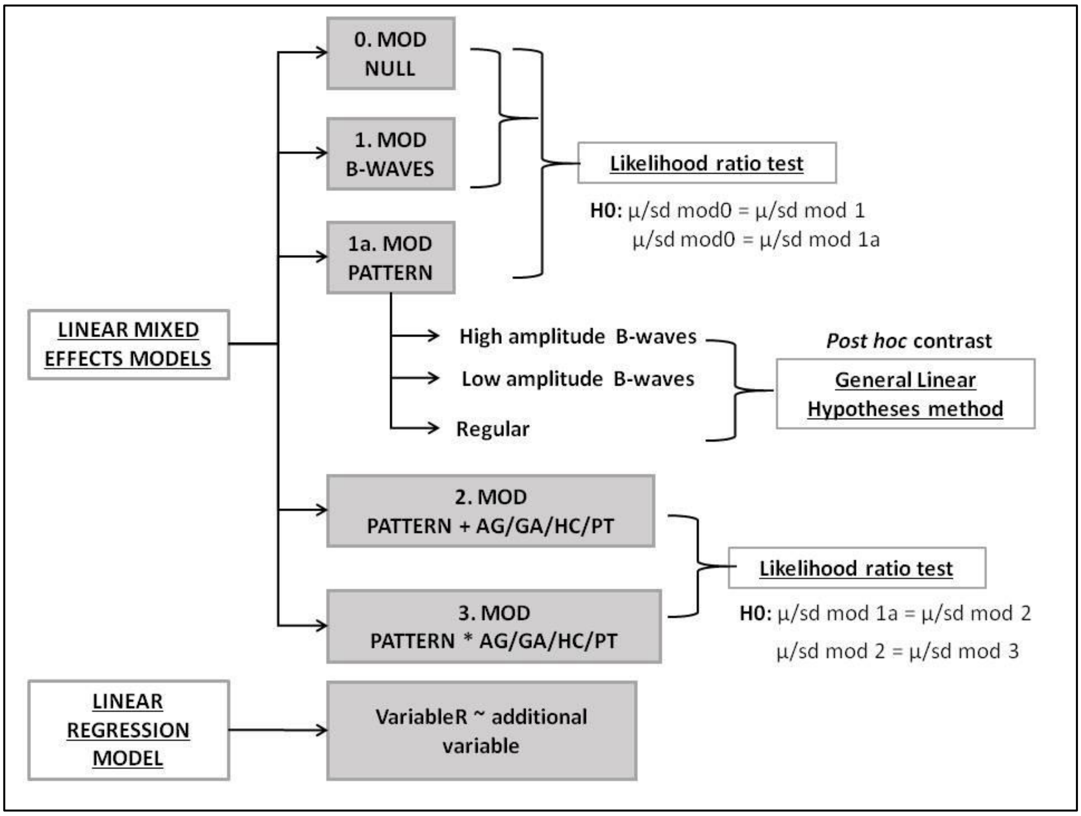

Supplemental figure 1. Statistical analysis. The algorithm used to perform the statistical analysis is shown. **MOD**: model; **H0**: null hypothesis; **μ**: mean; **sd**: standard deviation; **AG**: age; **GA**: gestational age; **HC**: head circumference; **PT**: probe type; **VariableR**: variable during regular pattern.

2

The R script used for studying the changes of cerebral hemodynamics during ICP B-waves is shown below. As first step we have built the model.

```
modICP0 <- lmer(ICP ~ 1  + (1|Subject.ID), data=LME.frame, REML=FALSE )
summary(modICP0)
modICP1 <- lmer(ICP ~ 1 + Waves+ (1|Subject.ID), data=LME.frame, REML=FALSE )
summary(modICP1a)
```

```
modICP1a <- lmer(ICP ~ 1 + Pattern + (1|Subject.ID), data=LME.frame, REML=FALSE )
summary(modICP1a)
```

Then we have performed the likelihood ratio test:

```
anova(modICP1a,modICP0)
anova(modICP1a,modICP0)
```

We have checked the residuals for significant models:

```
plot(fitted(modICP1a), residuals(modICP1a))
hist(residuals(modICP1a))
qqnorm(residuals(modICP1a))
```

For the model including patterns a post hoc contrast analysis was performed for each parameter using the general linear hypotheses (GLH) method.

```
glht(modBFI1,linfct = mcp(Pattern="Tukey"))
summary(glht(modBFI1,linfct=mcp(Pattern="Tukey")))
```

We have conducted this analysis both with the mean and the standard deviation values of the clinical and optical variables. We have then studied the effect of demographic and clinical variables on cerebral hemodynamics; the R script is shown below. We have checked the effect of age in months, gestational age (GA), head circumference (HC) and probe type (PT) by building additional linear mixed effects (LME) models.

```
modICP2<-lmer(ICP ~ 1 + Pattern + Age/GA/HC/PT + (1|Subject.ID), data=LME.frame, REML=FALSE )
summary(modICP2)
modICP3<-lmer(ICP ~ 1 + Pattern * Age/GA/HC/PT + (1|Subject.ID), data=LME.frame, REML=FALSE )
summary(modICP3)
anova (modICP1a,modICP2)
anova (modICP2, modICP3)
```

We have also investigated the association of the cerebral hemodynamics variables (THC, StO_2, CBF) with demographic and clinical parameters such as psychomotor delay, presence of symptoms, prematurity, gender, macrocephaly. To do so we have considered the mean values of THC, StO_2, CBF during the regular period. We have built a linear regression model.

```
Mod4<-lm(variableR ~1,data=Descriptive, REML=FALSE )
summary(mod4)
```

```
mod5<-lm(variableR ~ psychomotor delay/symptoms/prematurity/gender/macrocephaly, data=Descriptive, REML=FALSE )
summary(modICP5)
anova(modICP4,modICP5)
```
